# Benchmarking Whole‐Body Controllers on the TALOS Humanoid Robot

**DOI:** 10.3389/frobt.2022.826491

**Published:** 2022-03-04

**Authors:** N. Ramuzat, O. Stasse, S. Boria

**Affiliations:** ^1^ LAAS-CNRS, University of Toulouse, Toulouse, France; ^2^ Airbus, Toulouse, France; ^3^ Artificial and Natural Intelligence, Toulouse Institute, Toulouse, France

**Keywords:** humanoid robot, benchmarking, whole body control, balance, torque control

## Abstract

This article presents a comparison of three control schemes applied on the commercially available TALOS humanoid robot. The aim is to highlight the advantages and drawbacks of each model applied on three locomotion problems: walking on flat and non-flat terrain and climbing stairs. The different models are based on position control (first and second models) or torque control (third model). The first one uses a hierarchical quadratic program at velocity level. The second one uses a weighted quadratic program named Task Space Inverse Dynamic (TSID) at acceleration level. Finally, the last one also uses TSID but at torque level. The controller performances are compared in simulation, using Gazebo, on the accuracy of their tracking, their energy consumption, and their computational time execution.

## 1 Introduction

### 1.1 Goal

Bipedal locomotion of humanoid robots is considered a difficult problem because of the complexity of robot dynamics, the numerous constraints of the motion, and the unknown environment. The design choice made when designing a robot may have a strong impact on the control laws that are really working on the system and the real performances. A recent example is the Digit robot which has very impressive capabilities by choosing a careful tradeoff between the chosen actuation technology and robot weight distribution Robotics (2022). The robot is very robust to impact and allows torque control but is slightly limited by the payload it can carry (10 kg). Realizing torque control on electric-based bipedal system is challenging. If it was successfully realized on the TORO robot Englsberger et al. (2014) for standing whole body control and walking, it is notoriously more difficult to achieve than position control. A striking example is given by the iCub robot with which impressive Tai chi motions have been realized Pucci et al. (2016) but where walking in torque control mode is still difficult to achieve Romualdi et al. (2019). The goal of this study is to report a similar evaluation with the commercially available TALOS robot from PAL-Robotics.

### 1.2 Motion Execution Pipeline

Three stages are usually considered to execute a motion on a humanoid robot: the contact sequence generation, trajectory planning, and whole-body control.

Most of trajectory planning methods use centroidal dynamics to generate consistent behaviors for a legged robot. In this study, we use preplanned trajectories provided either by a standard walking pattern generator or by a multicontact planner Fernbach et al. (2020) The latter is used for a platform which can be easily rebuilt for benchmarking walking on uneven terrain. This planner provides a centroidal trajectory that is dynamically balanced on uneven terrain and does not assume that the robot behaves completely like a Linear Inverted Pendulum (LIPM). Because the centroidal dynamics is planned and the setup limits the number of contacts to one or two, it is still possible to apply the concept of Divergent Component of Motion (DCM) Takenaka et al. (2009); Englsberger et al. (2015) for control. The newly generated reference DCM is used for admittance control on the Center of Mass (CoM) as given by Caron et al. (2019); Romualdi et al. (2019).

Then, to track the reference trajectories, a whole-body controller is needed. Whole-body controllers are based on the task function approach Samson et al. (1991); Escande et al. (2014) from which a quadratic program is formulated. Complex motions combine several nonlinear tasks and constraints. In this study, two types of QP formulations are compared, a hierarchical QP which imposes a strict hierarchy between the tasks Henze et al. (2016); Herzog et al. (2014) and a weighted QP which sets weights to prioritize the tasks Koolen et al. (2016); Cisneros et al. (2018).

In the recent literature, there is a growing number of implementations of torque-based whole-body control algorithms Koolen et al. (2016); Herzog et al. (2014); Lee and Goswami (2012); Englsberger et al. (2015). Indeed, due to the intrinsic compliance of the torque control formulation, it is more suitable for interactions with humans and for multicontact problems where external interactions and several contact points are needed. However, the transition from the simulations to the real experiments is harder due to inaccuracies on the actuation chain model Ramuzat et al. (2020). Such inaccuracies do not appear when using position control.

### 1.3 Contributions

**FIGURE 1:**
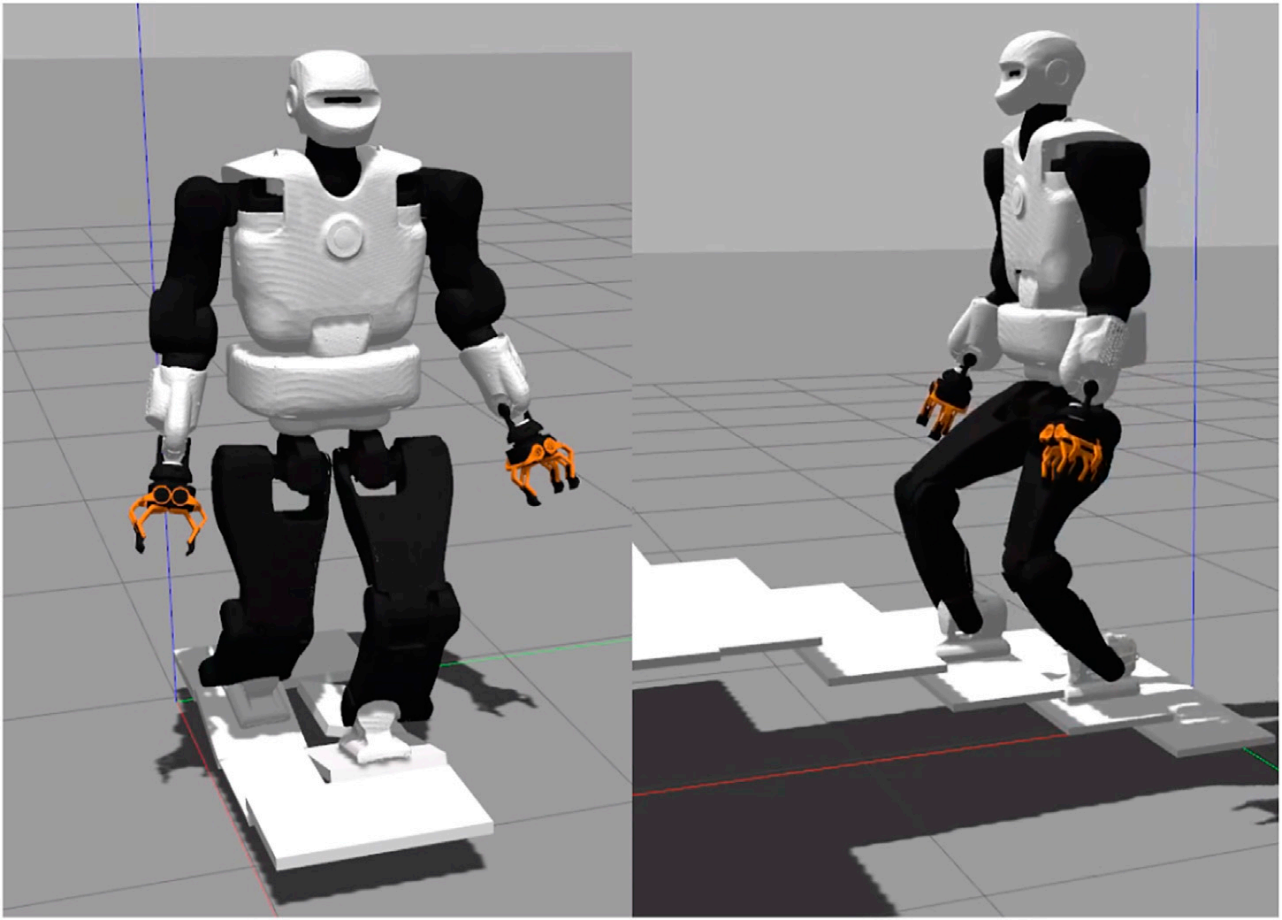
Walking on tilted platforms and climbing stairs.

Following the existing benchmarking of humanoid robot control architectures Romualdi et al. (2019); Stasse et al. (2018), this study contributes by benchmarking the TALOS humanoid robot. It is performed by comparing three whole-body control schemes on the TALOS robot in simulation. Two are using position control associated with DCM and CoM admittance controls and one is using torque control. The first one is based on a lexicographic QP using inverse kinematics (denoted *IK* in this article), while the second and the third one use a weighted QP (WQP) with inverse dynamics and an angular momentum (AM) task (denoted respectively *TSID position* and *TSID torque*). They are evaluated in Gazebo simulations on three locomotion problems: walking on flat, uneven terrains, and stairs (Figure 1), on the criterion of trajectory tracking, energy consumption, passivity, and computational cost. As a first consequence of our torque control scheme, we achieve the highest walking velocity for the robot TALOS in simulation: 0.6 m/s. We believe that the motion on an uneven terrain with the platforms is novel and offers an interesting new benchmark. Finally, we also provide an evaluation of the passivity gait measure that we believe is interesting to measure the efficiency of a balance strategy in terms of energy.

We organize the article as follows: Section 2 recalls the centroidal dynamics equations, DCM control, and AM task. Section 3 details the three task-space whole-body control schemes compared in this study. Section 4 presents the energy criterion used. Section 5 describes the planning methodologies used to obtain the reference trajectories for the simulations. Then, Section 6 presents these simulations results, and Section 7 describes the experiments achieved on TALOS and their limitations.

## 2 Centroidal Dynamics

The under-actuated part of the robot whole-body dynamics is called centroidal dynamics. It uses the Newton–Euler equations of motion which couple the variations of the centroidal momentum with contact forces Orin et al. (2013):

$$\begin{array}{ccc} \left\{\begin{array}{cc} m\ddot{c}= & \underset{i}{\sum }f_{i}+mg={\dot{l}}_{c}\\ mc_{\times }\left(\ddot{c}-g\right)+\dot{L}= & \underset{i}{\sum }\left(p_{i}-c_{i}\right)\times f_{i}+\tau_{i}={\dot{k}}_{c}, \end{array}\right. & & \end{array}$$ (1)

with $c,\dot{c},\ddot{c}$ the CoM position *c* = (*c*_*x*_, *c*_*y*_, *c*_*z*_), velocity and acceleration, $\dot{L}=\sum_{k}\left[R_{k}I_{k}{\dot{w}}_{k}-R_{k}{\left(I_{k}w_{k}\right)}_{\times }w_{k}\right]$ , and $g={\left[0,\;0,\;-9.81\right]}^{T}$ , where *R*_*k*_ ∈ *SO*(3) is the 3d rotation matrix between the *kth* body frame and the inertial coordinate frame, *I*_*k*_ its inertial matrix, *w*_*k*_ its angular velocity, *m* is the mass of the robot, $f_{i}\in R^{3}$ the vector of contact forces at contact point *i*, $p_{i}\in R^{3}$ their positions, and $\tau_{i}\in R^{3}$ their contact torque (represented at the inertial coordinate frame). *l*_*c*_ and $k_{c}\in R^{3}$ are the linear and angular momentum around the CoM and

$$\begin{array}{ccc} c_{\times }=\left(\begin{array}{ccc} 0 & -c_{z} & c_{y}\\ c_{z} & 0 & -c_{x}\\ -c_{y} & c_{x} & 0 \end{array}\right). & & \end{array}$$

### 2.1 Divergent Component of Motion

We use the DCM formulation for the admittance control of the CoM. Under the assumptions of the LIPM, we can obtain the following set of equations Takenaka et al. (2009); Englsberger et al. (2015):

$$\begin{array}{cc} \dot{c}= & \omega \left(\xi -c\right)\\ \dot{\xi }= & \omega \left(\xi -z\right)\\ \xi = & c+\frac{\dot{c}}{\omega } \end{array},$$ (2)

with *z*, *ξ* respectively the zero moment point (ZMP) and DCM and $\omega =\sqrt{g/c_{z}}$ . These equations show that the DCM is the divergent component of the LIPM model. Thus, the DCM needs to be controlled to stabilize the system Kajita et al. (2010); Sugihara (2009); Englsberger et al. (2015); Mesesan et al. (2019). Caron et al. (2019) propose to use a proportional–integral (PI) control on the DCM (the integral term is used to eliminate the steady-state error). Romualdi et al. (2019) propose an asymptotical criterion, but other techniques which guarantee stability can be used.

In terms of ZMP, the obtained control law is as given by Caron et al. (2019):

$$\begin{array}{ccc} z^{*} & = & z^{ref}-\left[1+\frac{kp_{\mathrm{dcm}}}{\omega }\right]\left(\xi^{ref}-\xi \right)\\ & & +\frac{kz_{\mathrm{dcm}}}{\omega }\left(z^{ref}-z\right)-\frac{ki_{\mathrm{dcm}}}{\omega }\int \left(\xi^{ref}-\xi \right)dt \end{array},$$ (3)

with *z* ^*ref*^, *ξ* ^*ref*^ the respective ZMP and DCM reference values, given by the planning. Finally, this desired ZMP is used into a CoM admittance control as found in the study by Caron et al. (2019):

$${\ddot{c}}^{*}={\ddot{c}}^{ref}+kp_{\mathrm{adm}}\left(z-z^{*}\right).$$ (4)

**FIGURE 2:**
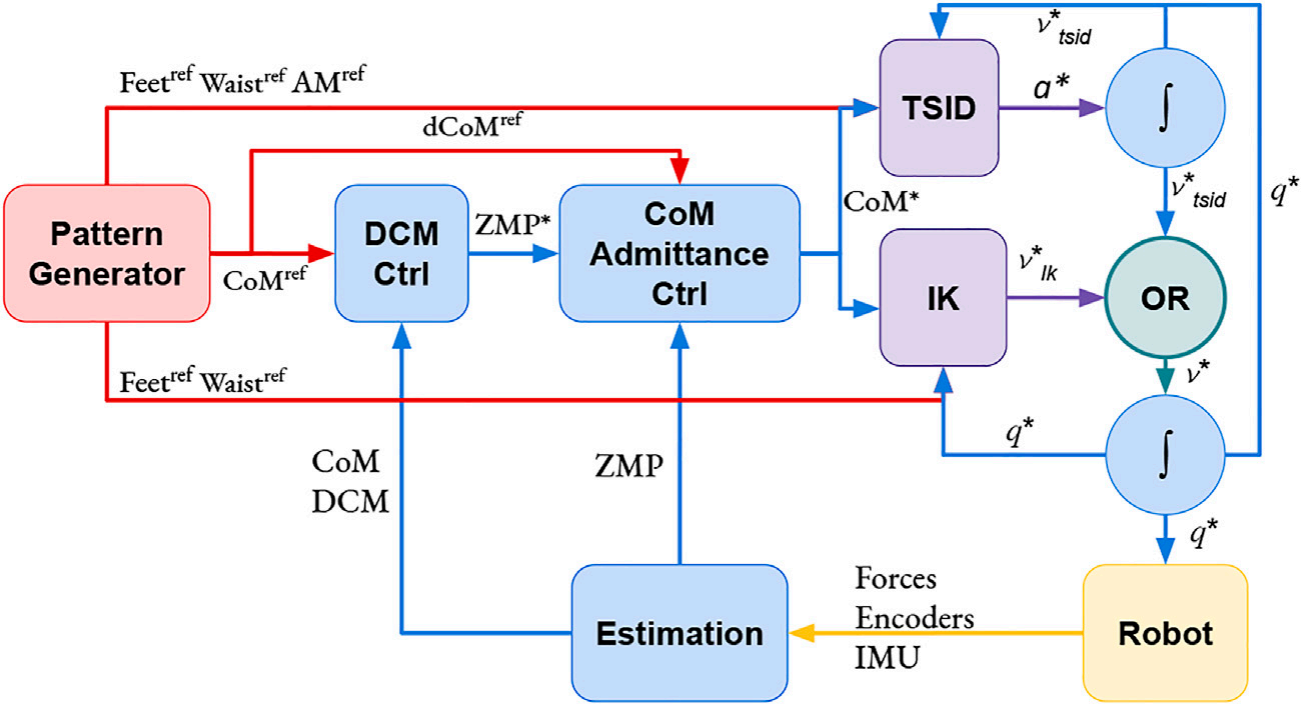
Position control schemes: IK and TSID. The *OR* block is used to activate only one controller at a time.

The two position control schemes presented in this study use this stabilization formulation. In Figure 2, Eq. 3 is implemented in the *DCM Ctrl* blue block and Eq. 4 in the *CoM Admittance Ctrl* one. See Table 1 for the gains value used in the simulations.

**TABLE 1:** Task gains of the control schemes, tilted platforms, and stair simulations use the same gains.

Task gains	IK	TSID position	TSID torque
	(20 cm∣stairs)	(20 cm∣stairs)	(20–60 cm∣stairs)
*K*_p*com*_	100	1,000	20∣12
*K*_d*com*_	—	300	3
*K*_p*comH*_	100	1,000	—
*K*_d*comH*_	—	300	—
*K*_p*waist*_	300	100	100
*K*_d*waist*_	—	20	20
*K*_p*contacts*_	1,000	30	30–100∣30
*K*_d*contacts*_	—	11	11–0∣11
*K*_p*feet*_	1,000	2000	1,200∣500
*K*_d*feet*_	—	20	12
*K*_p*am*_	—	10	10
*K*_p*posture*_	100	see below	see below
*K*_d*posture*_	—	$2\sqrt{K_{\text{p}posture}}$	$2\sqrt{K_{\text{p}posture}}$
*K*_p*comAdm*_	15∣45	12	—
*K*_p*dcm*_	8∣25	8	—
*K*_i*dcm*_	1	1	—
*K*_z*dcm*_	1	1	—
**TSID Gains**	**legs**		**torso**
*K*_p*posture*_	(10, 5, 5, 1, 10, 10)		(100, 100)
—	arms		head
*K*_p*posture*_	(50, 10, 10, 10, 50, 10, 10, 10)		(100, 100)

### 2.2 Centroidal Momentum Tasks

The objective is to consider the angular momentum part of the Euler equation generated by the contact transition (Kajita et al. (2003b). Using equation Eq. 1, the centroidal dynamics is, therefore, defined by $h_{c}={\left[l_{c}\;k_{c}\right]}^{T}\in R^{6}$ . In the study by Wensing and Orin (2013), the task formulation of the centroidal dynamics control is given by $h_{c}=A_{G}\left(q\right)\dot{q}$ , where $q,\dot{q}$ are the joint position and velocity vectors of the robot and *A*_*G*_ is the centroidal momentum matrix Orin et al. (2013).

The tasks dynamics are given by the following equations:

$$\left\{\begin{array}{cc} {\dot{l}}_{c}= & m\left[{\ddot{c}}^{*}+K_{\text{D}com}\left({\dot{c}}^{*}-\dot{c}\right)+K_{\text{p}com}\left(c^{*}-c\right)\right]\\ {\dot{k}}_{c}=\; & {\dot{k}}_{c}^{*}+K_{\text{p}am}\left(k_{c}^{*}-k_{c}\right) \end{array}\right..$$ (5)

The angular momentum task in the TSID is expressed as in equation Eq. 5 and successfully implemented in the study by Lee and Goswami (2012) (the gains are defined in Table 1).

## 3 Whole-Body Controller

### 3.1 Lexicographic Quadratic Programming

The first controller used is a lexicographic QP task-based inverse kinematics described by Mansard et al. (2009). In this controller, the task errors *e* to be reduced in the cost function are implemented as velocity-based tracking laws in the Lie group SE(3). Having the robot configuration vector *q* and the joint velocity $\dot{q}$ as control input, a task-function is a derivable function *x*(*q*) whose space is named task space. In addition, the task errors *e* are expressed as follows:

$$\begin{array}{ccc} \dot{e}\left(q,t\right) & = & \dot{x}\left(q\right)-{\dot{x}}^{*}\left(t\right)\\ \dot{x}\left(q\right) & = & J\dot{q} \end{array},$$ (6)

with $J=\frac{\partial e}{\partial q}=\frac{\partial x}{\partial q}$ the Jacobian according to the robot state vector.

The following dynamics is imposed on these errors:

$$\begin{array}{cccc} & \dot{e}\left(q,t\right) & = & K_{P}\left(x\left(q\right)⊖x^{*}\left(q\right)\right)\\ \Leftrightarrow & \dot{x}\left(q\right) & = & {\dot{x}}^{*}\left(t\right)+K_{P}\left(x\left(q\right)⊖x^{*}\left(q\right)\right) \end{array},$$ (7)

with ⊖ the difference operator of the Lie group.

*Inverse Kinematics QP: IK*—This control scheme is based on a DCM controller Eq. 3, a CoM admittance controller, Eq. 4 and a lexicographic QP solving the inverse kinematics of the robot (Figure 2). The authors have implemented this scheme in an open-source package GEPETTO Team LAAS-CNRS (2021c) based on the QP in the study by Mansard et al. (2009) adding the DCM and CoM admittance controllers.

The tasks used during the simulations are (the priority 0 is the highest one):

• Feet tracking (priority 0)
• CoM height tracking (priority I)
• CoM lateral–sagittal tracking (priority II)
• Waist orientation (priority III)
• Posture regularization in half-sitting (priority IV)

The respective task gains are defined in Table 1.

### 3.2 Task Space Inverse Dynamics

TSID Del Prete (2021) is a WQP which sums the task functions in a general cost function using weights to define their priorities (as opposed to the *IK* controller, it is not a strict hierarchy; it has only two strict layers: the constraint and the cost). In this controller, the task errors *e* to be reduced are implemented as acceleration-based tracking laws in the task space. Having the robot configuration vector *q* and the joint acceleration $\ddot{q}$ as control input, a task function is a second-order derivable function *x* of *q*. In addition, the task errors *e* are expressed as follows:

$$\begin{array}{ccc} \ddot{e}\left(q,t\right) & = & \ddot{x}\left(q\right)-{\ddot{x}}^{*}\left(t\right)\\ \ddot{e}\left(q,t\right) & = & \left(J\ddot{q}+\dot{J}\dot{q}\right)-{\ddot{x}}^{*}\left(t\right) \end{array}.$$ (8)

The following dynamics is imposed on these errors:

$$\begin{array}{cccc} & \ddot{e}\left(q,t\right) & = & K_{P}\left(x\left(q\right)⊖x^{*}\right)+K_{D}\left(\dot{x}\left(q\right)-{\dot{x}}^{*}\left(t\right)\right)\\ \Leftrightarrow & \ddot{x}\left(q\right) & = & {\ddot{x}}^{*}\left(t\right)+K_{P}\left(x\left(q\right)⊖x^{*}\left(t\right)\right)+\\ & & & K_{D}\left(\dot{x}\left(q\right)-{\dot{x}}^{*}\left(t\right)\right) \end{array}.$$ (9)

TSID solves the inverse dynamics of the robot in rigid contact with the environment Herzog et al. (2014) and has been successfully used on the HRP-2 robot in the study by Del Prete et al. (2016).

*Inverse Dynamics WQP: TSID Position*—This control scheme is based on a DCM controller Eq. 3, a CoM admittance controller Eq. 4 and a WQP solving the inverse dynamics of the robot, Figure 2. Compared to the previous controller, this one implements an AM task, which regulates the angular momentum to 0, using the formulation of Eq. 5. The authors have implemented this controller using TSID Del Prete (2021) library in the same package than the controller *TSID Torque* with the DCM and CoM admittance controllers.

The tasks considered during the simulations are as follows:

• Feet tracking (priority 0)
• Feet contacts (priority 0)
• CoM height tracking (priority I, weight 10^3^)
• CoM lateral-sagittal tracking (priority I, weight 10^3^)
• Waist orientation (priority I, weight 1)
• Posture regularization in half-sitting (priority I, weight 0.1)
• AM velocity-acceleration regularization (priority I, weight 2 × 10^–2^)

The respective task gains are defined in Table 1. The weights and gains have been chosen through trials and errors with an a priori heuristic.

**FIGURE 3:**
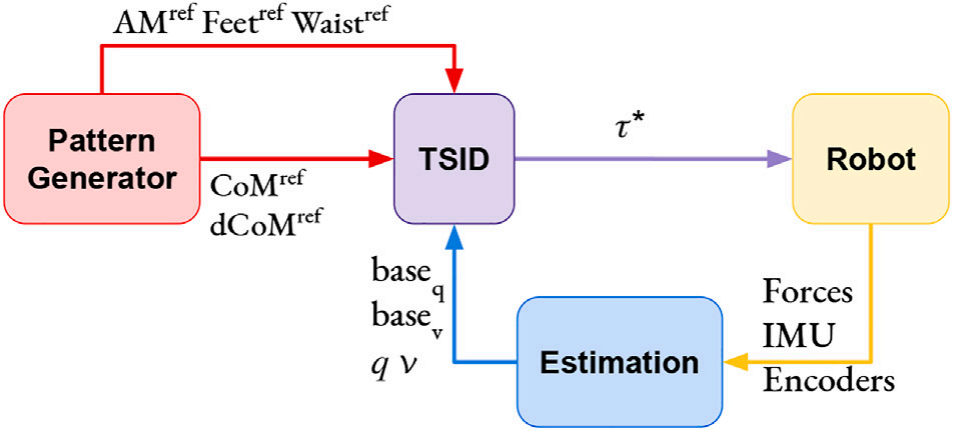
TSID torque control scheme.

*Inverse Dynamics WQP: TSID Torque*—This control scheme is based on a WQP solving the inverse dynamics of the robot (with an AM regularization task, using the formulation of Eq. 5), as shown in Figure 3. From the desired acceleration computed by the QP, TSID retrieves the associated torque by using the robot equation of the dynamics. The authors have implemented this controller using the TSID Del Prete (2021) library in the open-source package GEPETTO Team LAAS-CNRS (2020).

The tasks considered in the simulations are the same as *TSID position*, with different gains (Table 1).

### 3.3 Remark on the State Feedback

For the position control, it is needed to integrate the result of the QP (one time for *IK* and two times for *TSID position*, Figure 2) to obtain the desired command. To avoid instabilities, the control loop of both QPs use these integrated values in the next iteration instead of the measured ones. The measured position and velocity of the robot are only used to compute the CoM, DCM, and ZMP for the admittance control in the position schemes. On the contrary, the torque control scheme uses the measured values at each iteration of the QP (Figure 3) and, in particular, the position and velocity of the robot base (or free-flyer).

## 4 Energetic Comparison Criterion

### 4.1 Energy Cost

Based on the study by Torricelli et al. (2015), a relevant criterion to compare the energy consumption of the control schemes is the cost of transport. It can be computed as the energetic cost of transport *C*_*et*_ using the whole mechanical work of the actuation system *E*_*m*_ or as the mechanical cost of transport *C*_*mt*_ using only the positive one *E*_*m*+_.

$$C_{et}=\frac{E_{m}}{mgD},\;C_{mt}=\frac{E_{m+}}{mgD},\;E_{m}=\int_{0}^{T}\overset{N}{\underset{i=0}{\sum }}|\tau_{i}\left(t\right)\;\omega_{i}\left(t\right)|dt,\;E_{m+}=\int_{0}^{T}\overset{N}{\underset{i=0}{\sum }}\varrho_{i}\left(t\right)dt,\text{ if }\varrho_{i}\left(t\right)>0,$$ (10)

with *m* the mass of the system, *g* the gravity constant, *D* the distance traveled by the system, and *τ*_*i*_, *ω*_*i*_ the respective torque and velocity of each robot joint for all (*N*) joints and *ϱ*_*i*_(*t*) = *τ*_*i*_(*t*)*ω*_*i*_(*t*).

### 4.2 Passivity Gait Measure

Another interesting energetic criterion is the ability to minimize joint torques to increase the passivity of the walk Torricelli et al. (2015). The passivity gait measure (PGM) Mummolo and Kim (2012) quantifies the passivity of a biped walking motion:

$$\begin{array}{ll} PGM & =1-\frac{RMS\left(\tau_{sa}\right)}{RMS\left(\tau_{tot}\right)} \end{array}$$ (11)

$$\begin{array}{ll} RMS\left(\tau_{tot}\right) & =\sqrt{\frac{\int_{0}^{T}\left[\sum_{i=0}^{N}\tau_{i}{\left(t\right)}^{2}\right]dt}{T}}, \end{array}$$ (12)

where *RMS* is the root mean square along the period of time *T*, *τ*_*sa*_ stands for the torque on the stance ankle joint, and *τ*_*tot*_ for the torque on all robot joints.

## 5 Locomotion Planning

### 5.1 Walking Pattern Generator

The trajectories used in the straight walk simulations have been computed using the algorithm described in the study by Kajita et al. (2003a); Stasse et al. (2008); GEPETTO Team LAAS-CNRS (2021a). This algorithm provides desired trajectories for the ZMP z*, the CoM c*, and the feet $p_{i}^{*}$ for a given set of footsteps (pre-defined in these simulations). This implementation uses the centroidal dynamics and dynamic filter proposed in the study by Kajita et al. (2003a) computed with the recursive Newton–Euler algorithm Featherstone (2008) implemented in the Pinocchio library Carpentier et al. (2019). The CoM trajectory is modified to take into account the momentum generated by the limb motion. The desired DCM ξ* is deduced from the desired CoM c* and desired ZMP z* trajectories (Eq. 2).

### 5.2 Multicontact Locomotion Planning

The trajectories used in the tilted platform and stair simulations have been computed using the open-source framework multicontact locomotion planning GEPETTO Team LAAS-CNRS (2021b). Given the initial and final poses of the robot, the framework computes a reachability plan and a contact sequence as given by Tonneau et al. (2020). Then, it optimizes the centroidal dynamics (Section 2) using two convex relaxations based on trust regions Ponton et al. (2018). Similar to the pattern generator method, it takes into account the momentum generated by the swing leg owing to iterations between a kinematic whole-body formulation and the centroidal dynamic optimization. In contrast, when solving Eq. 1, it does not assume that $\dot{L}=0$ (Section 2).

## 6 Simulation Results

The simulations realized in this study have been made using Gazebo. A video illustrating the simulations is available at the following link: https://peertube.laas.fr/videos/watch/4b5d3a5b-2355-47a0-8197-f41ed4f885c6. The chosen simulations are walking on flat or uneven terrains and climbing stairs. Based on the study by Torricelli et al. (2015), they cover different aspects of locomotion skills for a stationary environment with and without unexpected disturbances.

### 6.1 Straight Walk of 20-cm Steps

**FIGURE 4:**
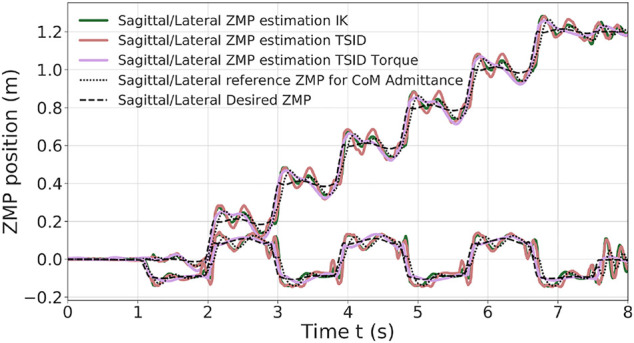
ZMP estimation of the 20-cm step walk.

**FIGURE 5:**
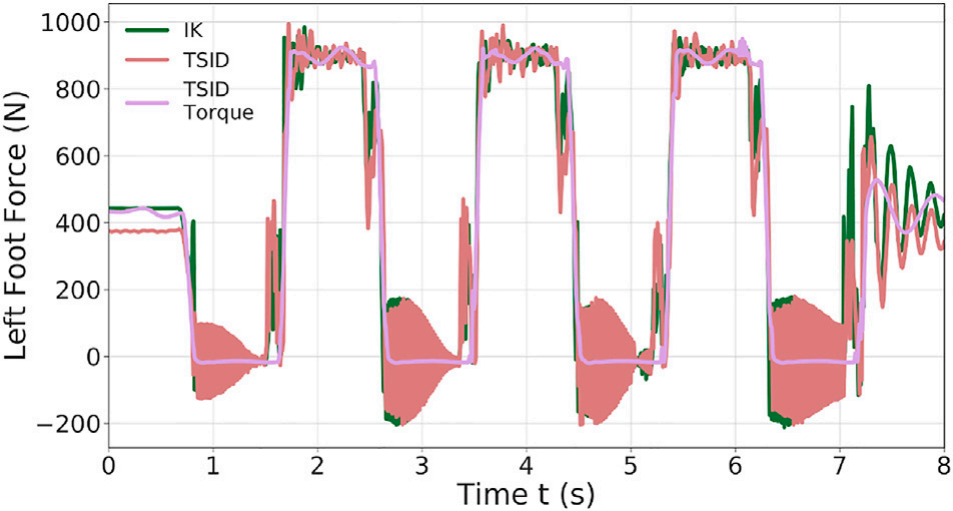
Z-axis left foot force of the 20-cm step walk.

In the simulation, the robot executes six steps forward at 0.2 m/s and a final step (traveled distance of 1.2 m). The time distribution is 0.9 s for single-support phase and 0.115 s for double-support phase (leading to steps of approx. 0.20 m). The controllers have also been successfully tested on a faster walk with single/double support time of 0.711/0.089 s. Figure 4 presents a comparison of the three control schemes on their estimated ZMP on the sagittal (x-axis, top curves on the figure) and lateral (y-axis, bottom curves) planes only because the desired height of the CoM is constant. Figure 5 shows the forces applied on the ground along the z-axis on the left foot. The tracking of the CoM and feet is accurately followed by the three controllers (tracking error lesser than 1 cm).

The two position controllers achieve similar results, tracking correctly the ZMP reference of Eq. 3, with an average error of 2 cm (Table 2). Noticeably, the torque control presents a ZMP which is close to the position control results in Figure 4, even though there is no explicit control on the ZMP or the DCM. In the Tables presenting the error on the ZMP, for the torque scheme, the estimated ZMP is compared to the desired ZMP (from the planning). In particular, in the lateral plane, the error is quite low, 1 *cm* in average.

**TABLE 2:** ZMP error of the 20-cm step walk simulation.

Control scheme	Axis	Average	Standard deviation	Peaks (m)
*IK*	x-axis	0.019 m	0.022 m	0.131
	y-axis	0.022 m	0.026 m	0.150
*TSID*	x-axis	0.028 m	0.025 m	0.142
*position*	y-axis	0.025 m	0.027 m	0.138
*TSID*	x-axis	0.026 m	0.021 m	0.078
*torque*	y-axis	0.011 m	0.014 m	0.078

Figure 5 illustrates the ground impact problem in the position control compared to the better foot landing observed in torque control. Indeed, each time the left foot comes into contact with the ground (1.5, 3.5 s, … ), the *IK* and *TSID position* schemes show peaks in the foot force ( ∼400 N) which are avoided in *TSID torque*. This also explains the peaks in the ZMP errors (around 15 cm) because during an impact, the foot bounces on the ground. The force oscillations of the *IK* and *TSID position* controllers when the foot is in the air are due to the high control gains on the ankle (proportional–integral–derivative (PID) gains of the low-level position control in Gazebo); it is mainly noises.

### 6.2 Straight Walk of 60-cm Steps in Torque Control

In the study by Mesesan et al. (2019) the humanoid robot TORO successfully performed a walk on flat terrain with a step length of 55 cm (single/double support time of 1.1/0.4 s). In the following simulation, the torque controller is pushed to its limits to show its capability to achieve a similar result. The robot TALOS executes six steps forward of 0.6 m/s and a final one to go back to the initial position. The time distribution used is of 0.9 s for single-support phase and 0.115 s for double-support phase (leading to steps of approx. 60 cm).

**FIGURE 6:**
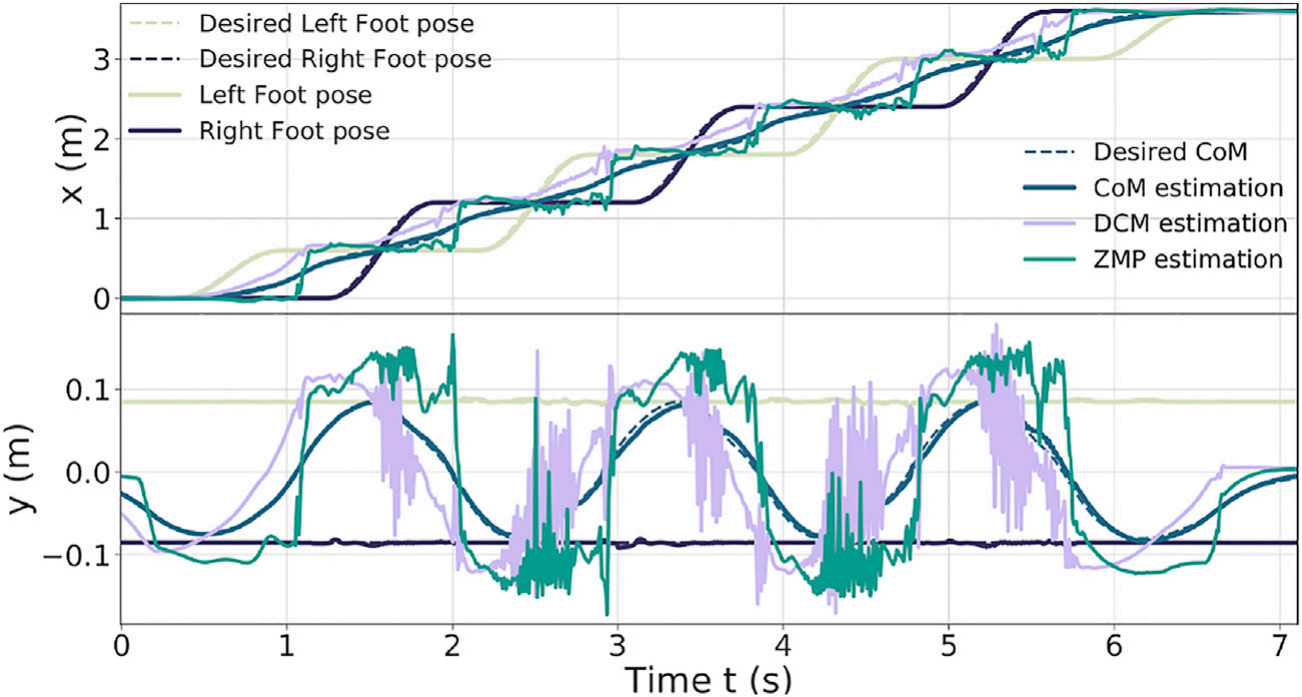
Feet, CoM, DCM, and ZMP of the 60-cm step walk.

Figure 6 presents the results obtained on the tracking of the feet and the CoM (Table 3); the ZMP and DCM estimations. The feet track well the desired trajectories along the y-axis (maximum error of 6 mm); however, along the x-axis, they show some delay (maximum error of 6 cm). Thus, it induces greater tracking errors on the x-axis for the CoM (peaks of 5 cm along the x-axis and 1.5 cm along the y-axis).

**TABLE 3:** CoM and feet error of the 60-cm step walk.

	Axis	Average	Standard deviation	Peaks (m)
CoM	x-axis	0.018 m	0.013 m	0.050
	y-axis	0.004 m	0.003 m	0.015
Left foot	x-axis	0.014 m	0.013 m	0.063
	y-axis	0.001 m	0.001 m	0.005
Right foot	x-axis	0.016 m	0.016 m	0.063
	y-axis	0.001 m	0.001 m	0.006

One can notice that the DCM and ZMP along the x-axis are more stable, whereas along the y-axis, they present large oscillations (which are caused by the feet impacts on the ground when landing).

**FIGURE 7:**
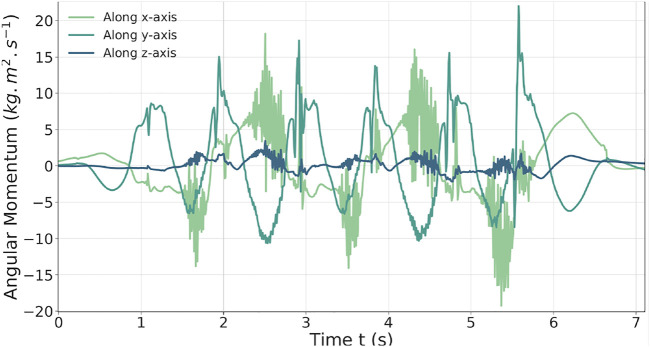
AM behavior during the 60-cm step walk in torque.

In Figure 7, the AM behavior is shown along the three axes. The AM task minimizes the momentum to zero. The x and y momentum components are the most solicited, leading to inclination of the torso forward and backward and to important moves of the arms to compensate the delay of the CoM and succeed the 60-cm steps. The authors observed that without this AM task, the walk cannot be achieved.

### 6.3 Walk on the Tilted Platforms: Uneven Terrain

In this third simulation, the robot walks on tilted platforms which represent uneven terrain (Figure 1). This walk is achieved using the multicontact locomotion planning trajectories (Section 5.2). The framework ensures the stability of the controllers on non-flat terrain when the feet are tilted.

**FIGURE 8:**
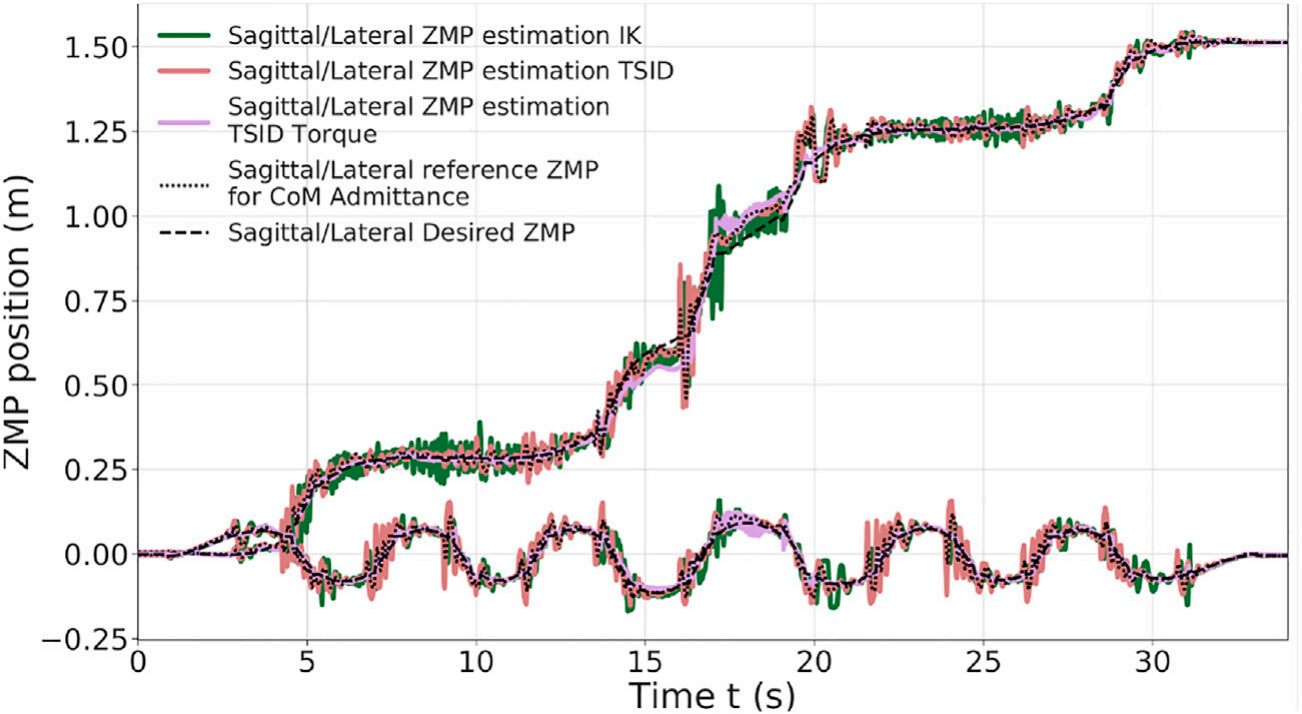
ZMP estimation of the tilted platform simulation.

Figure 8 illustrates the tracking performance of the controllers. The ones in position present the largest oscillations as *TSID torque* is the most stable (Table 4). Both the *IK* and torque control show oscillations at *t* ≈ 18 s; it corresponds to the worst case where the robot has its two feet tilted to keep its balance on two opposite platforms, leading to small slippages of the feet (this behavior can be observed in the linked video). These oscillations are larger in the case of the *IK* scheme. Similar oscillations on the contact forces in this part of the motion have also been observed, which are smaller in the case of the torque control. Increasing the gains on the feet only generates more instability, but raising the ones on the DCM and admittance control reduces the oscillations (at the cost of a more rigid behavior).

**TABLE 4:** ZMP error of the tilted platform simulation.

Control scheme	Axis	Average	Standard deviation	Peaks (m)
*IK*	x-axis	0.021 m	0.024 m	0.278
	y-axis	0.016 m	0.018 m	0.118
*TSID*	x-axis	0.012 m	0.017 m	0.197
*Position*	y-axis	0.015 m	0.019 m	0.127
*TSID*	x-axis	0.013 m	0.021 m	0.107
*Torque*	y-axis	0.005 m	0.006 m	0.058

Finally, the same result on the feet forces is obtained in this simulation with respect to the *20-*cm steps one. Due to the high gains on the DCM, to avoid the slippage of the robot, the *IK* control presents bigger peaks of force.

### 6.4 Climbing Stairs

**FIGURE 9:**
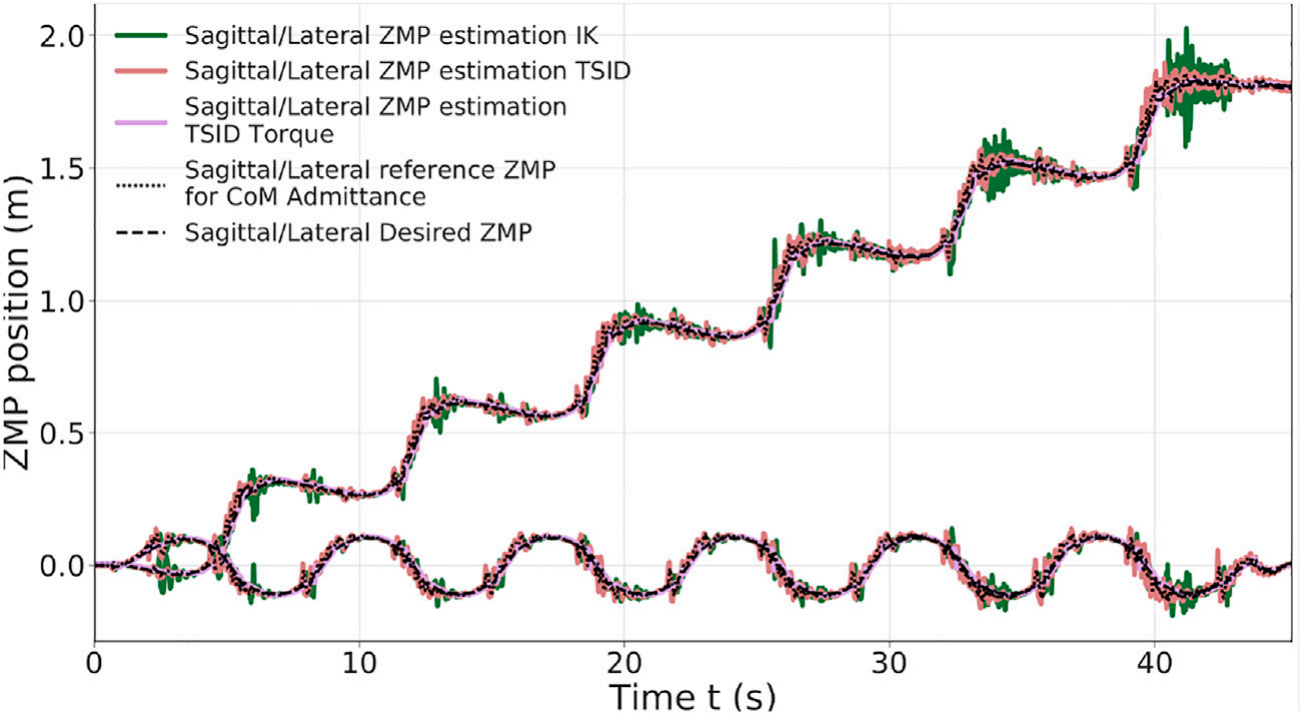
ZMP estimation of stair climbing.

In the last simulation, the robot is climbing six stairs of 10 cm height and 30 cm long (Figure 1). The trajectories are planned with the multicontact locomotion planning. Figure 9 shows the ZMP evolution of each controller, where the result is similar to the uneven terrai*n* simulation. The *TSID torque* scheme behaves significantly better than the others, with the ZMP matching the one planned (errors lesser than 1 cm, Table 5). Noticeably, the *IK* scheme presents higher oscillations at the end of the move in the lateral plane. The robot ends displaced on the right compared to the desired trajectories due to slippages of the feet when it finishes to climb a stair (shown in the linked video).

**TABLE 5:** ZMP error of the stair simulation.

Control scheme	Axis	Average	Standard deviation	Peaks (m)
*IK*	x-axis	0.022 m	0.026 m	0.257
	y-axis	0.015 m	0.017 m	0.151
*TSID*	x-axis	0.009 m	0.013 m	0.151
*Position*	y-axis	0.012 m	0.015 m	0.119
*TSID*	x-axis	0.008 m	0.006 m	0.049
*Torque*	y-axis	0.006 m	0.005 m	0.047

### 6.5 Energy Cost and Passivity Gait Measure

The results obtained for the cost of transport of the four simulations are presented in Table 6 depending on the control scheme. The results obtained for iCub by Romualdi et al. (2019) are also presented for comparison (computed using Eq. 10), as the human ones. The lower the energy consumption is, the better, and similarly, getting closer to the human cost of transport is an improvement.

**TABLE 6:** Results of the specific cost of transport.

Control	Simulation	*E*_*m*_	*E*_*m*+_	*C*_*et*_	*C*_*mt*_
Scheme	—	(J)	(J)	(J/kg/m)	(J/kg/m)
*Human*	—	—	—	0.2	0.05
*iCub*	—	—	—	—	—
*Position*	20 cm	—	—	—	0,49
*Torque*	20 cm	—	—	—	0.26
—	20 cm	1983.9	1,359.3	1.68	1.15
*IK*	Platforms	5418.7	3,769.2	3.7	2.6
—	Stairs	7,249.5	2,145.3	4.1	1.2
*TSID*	20 cm	2,324.5	764.1	1.97	0.65
*Position*	Platforms	5377.5	1,413.6	3.6	2.0
—	Stairs	6,812.6	2059.6	3.8	1.2
—	20 cm	521.8	259.3	0.44	0.22
*TSID*	60 cm	3,147.2	1,583.8	0.89	0.45
*Torque*	Platforms	1,378.6	668.5	0.93	0.45
—	Stairs	1861.1	1,205.5	1.1	0.68

Compared to the results obtained on iCub, the control in torque has a similar cost for the 20-cm step simulation. However, the cost of the position controllers presented in this article is higher because of their higher gains. The human efficiency is closer to the torque control, walking with a *C*_*et*_ around 0.2 *J*/*kg*/*m* Collins et al. (2005). Noticeably, the energy costs in torque for the tilted platforms and stair trajectories are still less important than the simpler walk in position; the *C*_*mt*_ never exceeds 1, even for the 60-cm walk. Overall, the controller *TSID position* consumes less energy than the *IK*.

The passivity gait measure comparison of the different simulations is reported in Table 7 for three gait stages: single support (single S. corresponding to the stance ankle), double support (double S.), and flying foot (flying F. where the foot has no contact with the ground). The human results are given as an indicator Mummolo and Kim (2012); the robot behavior is expected to be similar during double support and flying foot phase where the ankle should be passive.

**TABLE 7:** Results of the PGM on three gait stages.

	Simulation	Double S.	Single S.	Flying F.
*Human*	50 cm	1.0	0.6	∼1.0
—	20 cm	0.35	0.89	0.24
*IK*	Platforms	0.27	0.85	0.31
—	Stairs	0.46	0.86	0.36
*TSID*	20 cm	0.37	0.74	0.37
*Position*	Platforms	0.27	0.86	0.30
—	Stairs	0.55	0.86	0.34
—	20 cm	0.93	0.87	1.0
*TSID*	60 cm	0.87	0.79	1.0
*Torque*	Platforms	0.87	0.8	0.91
—	Stairs	0.97	0.89	1.0

The results of the position control schemes show a behavior which is the opposite of the human one. The passivity of the ankle is higher during the stance phase because of the control of the ZMP which minimizes ankle torque. In addition, it is weaker during the double support and flying phases due to the high PID gains of the low-level position control.

The control scheme in torque shows much more passive behavior (except on the stance foot) with a completely passive foot during the flying phase. During the double-support phase, the ankle is almost passive (*PGM*∼0.9), which is close to the human result. These results are better than the one expected in the study by Mummolo and Kim (2012), where the torque controlled robot has higher control on its stance ankle (*PGM* = 0.2).

Finally, on the uneven terrain, the double-support phase corresponds to the worst case where the robot has its two feet tilted to keep its balance on two opposite platforms. This leads to greater actuation than on flat floor (decreasing the passivity). Similarly, the stance phase corresponds to the left support phase on the final platform (highest slope), also leading to bigger actuation of the ankle.

### 6.6 Execution Time of the Control Schemes

The computational time obtained during the execution of one control loop of the three schemes is presented in Table 8, according to the simulations.

**TABLE 8:** Comparison of execution time.

Control scheme	Simulation	20 cm (60 cm)	Platforms (ms)	Stairs (ms)
*IK*	Average	0.5 ms	0.7	0.6
—	Peaks	2 ms	4	4
*TSID*	Average	1.2 ms	1.2	1.2
*Position*	Peaks	4.5 ms	4.3	4.2
*TSID*	Average	1 ms (1.4 ms)	1.2	1.1
*Torque*	Peaks	2.8 ms (6 ms)	5	5.5

The computational time of the *IK* is better due to computational efficiency of the null space projectors of the tasks. Exploiting this specific structure allows it to keep its control frequency higher than 1 kHz in average with four hierarchy levels. In *TSID*, this method can only be used once because it comprises two strict layers: the constraints and cost.

## 7 Experiments Realized on the Real Robot Using the Controllers

In this section are presented the results we succeeded to achieve on the real robot TALOS and the difficulties we encountered. These experiments are intermediate steps toward transferring the whole simulated results on the real robot. We detail the blocking points preventing us to successfully achieve these complete experiments.

### 7.1 TALOS Robot

Our robot TALOS is a humanoid robot of 1.75 m tall and about 100 kg comprising 32 joints and an under-actuated part called floating base (38 degrees-of-freedom in total). It provides the possibility to control the actuators in position control and torque control modes. It is performed owing to torque sensors on all the actuators, but the head and wrists. Humanoid robots often have flexible or compliant components. For instance, the actuator stiffness of the robot WALKMAN Negrello et al. (2017) can be directly tuned, creating an intended flexibility. Another example of the humanoid robot with compliant material is HRP-2 Nakaoka et al. (2007). It includes a bush rubber in the ankle to smooth impacts. In the robot TALOS, a non-intended flexibility on the hip link has been observed and impacts meaningfully the control of its legs and, therefore, its balance and locomotion. Indeed, this flexibility (not modeled in the simulator) leads to errors in the landing positions of the feet on the real robot. However, the deflection is not directly measurable by the encoders and cannot be directly modified.

### 7.2 Position Control

#### 7.2.1 Static Stabilization

**FIGURE 10:**
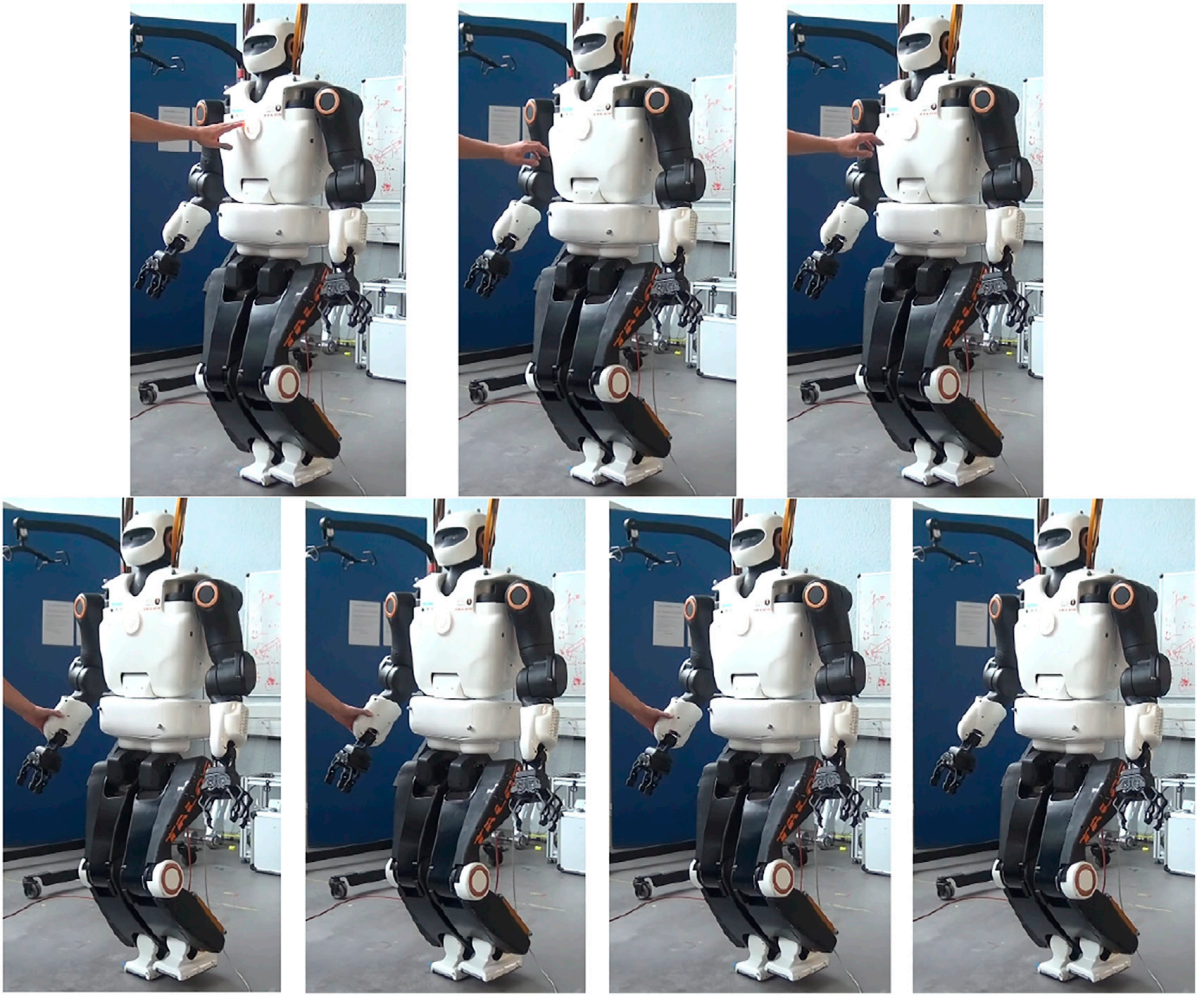
Experiments—push recovery of the TALOS robot with one foot raised.

Using the whole-body admittance control and the stabilizer described as the *IK* scheme in Section 3, the team achieved good results for balancing during quasi-static moves and standing position. Indeed, the admittance control at the CoM allows a quick reaction when applying external perturbations, such as pushing the robot. Figure 10 presents the reactive balancing of the TALOS humanoid robot when it is pushed from the front and from the side while standing on one foot. A video about this experiment is available at the following link: https://peertube.laas.fr/videos/watch/2dec7dba-cc57-4df4-8f10-a7d387404301.

In the video are shown push-recovery experiments while the robot is standing on both feet and with one foot raised. One can notice that the robot is more stable with both feet on the ground; nonetheless, the *IK* scheme allows a good stabilization at the CoM level. The stabilizer correctly achieves the balance of the robot: it controls the DCM such that the CoM does not diverge and applies correct contact wrenches to avoid falling (no slipping, not too much forces on one foot which imbalance the robot). It is important to underline that the admittance control is only implemented on the CoM; thus, the robot is stiff on its upper parts while more compliant on its lower parts (in particular, the hips and ankles). This is why in the video, pushing the robot arm produces motions on the whole robot and, in particular, its CoM.

The robot can achieve tasks with its upper body while external perturbations occur and keep its balance. It can also stabilize itself when non-dynamic trajectories are asked to the legs or with no contact with the ground (for instance, execute a swing on its foot). The difficulties appear with dynamic tasks involving the creation of contacts with the ground, typically during walking.

#### 7.2.2 Dynamic Stabilization

The dynamic stabilization of the robot is an ongoing work. The actual implementation of the stabilizer should allow the robot to achieve this goal; however, this is compromised by the flexibility in the hip of the robot TALOS. By tuning the gains of the admittance controller, the team manages to achieve once a straight walk of 20 cm using a WPG reference trajectories. The video of this success is available at the following link: https://peertube.laas.fr/videos/watch/b56d80ed-7c6c-46a7-8750-fdb7ea6d1636.

**FIGURE 11:**
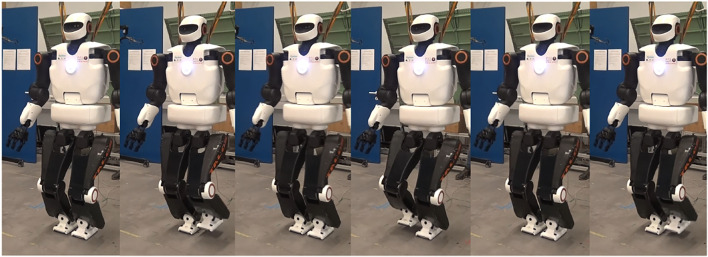
Experiments—on-spot walking with the TALOS robot.

Later on, we successfully achieved a repeatable on-spot walking which is quite stable (Figure 11). The video of this on-spot walking is available at the following link: https://peertube.laas.fr/videos/watch/1a920902-c75f-4fb0-a638-33bb9b48d649. One can notice that the left wrist of the robot is tilted; indeed, its absolute and relative encoders did not send the same value. Thus, when controlling its position, the wrist had an abnormal behavior as its returned position was not the good one. We had to deactivate its control for the experiment and then fix the offset of the relative encoder.

In both videos, the impacts on the ground are large and lead to instabilities, in particular slippage (which can also be caused by the flexibility in the hip). The robot has to move its upper body to compensate for them and because of that, at the end of the 20-cm walk, the robot almost falls. These impacts are partly due to the wrong positions of the feet when making contacts with the ground. The flexibility in each hip of the robot cannot be measured by the encoders; then, it creates an error between the positions given by the encoders and the real ones. These displacements at the hips are small, but when transferred at the feet positions, it can lead to errors of up to 5 cm. Thus, the controller is assuming a false position of the feet, and the robot enters in contact with the ground at a wrong position (even at the wrong moment, sooner if the displacement is in the direction of the walk or later in the opposite case). This creates large impacts and slippage, which prevents us to achieve successful walking; this is why compensating this flexibility is necessary. In the next subsection is presented the experiment realized to compensate it with a fixed value.

An additional way to cope with the stabilization problem would be to reschedule the footsteps and their location according to the landing time.

#### 7.2.3 Fixed Compensation of the Flexibility

We first try to compensate the flexibility by using a feedforward on the commanded position of the hip taking into account the torsional stiffness and measured torque. However, because of the noises on the torque sensors, we had to filter it which leads the compensation to be applied with delay. We also tried to activate this compensation only on single-support phases and not on double-support ones to avoid accumulation of internal efforts (on double support, the robot will try to correct its hip position while having its feet in contact with the ground and thus not moving, leading to this accumulation of energy). Even with such modifications, the results were not enough to successfully perform repeatable walk.

Thus, we then tried to impose a fix compensation of the flexibility without taking into account the measured torque. With a leg of 1 m weighting 20 kg, we fixed the compensation on the hip to $\Delta q^{hip}=\frac{20}{K}\approx \frac{20}{973}=0.021$ rad. Only a repeatable one step forward walk in position control has been successfully achieved with this method; see the video at the following link: https://peertube.laas.fr/videos/watch/08db3177-372b-43cc-85da-2009a267b5c9.

### 7.3 Torque Control

#### 7.3.1 PAL Robotics Low-Level Controller

To achieve torque control on the real robot, it is needed to transform the joint torque commands to motor currents. We decided to use the PAL robotics constructor low-level controller which computes new commands respecting the robot actuator dynamics. This low-level controller is a proprietary black-box, which uses a *ros-control* hardware interface to communicate with the robot. To interface our control scheme (based on the SoT with the WPG), we had to create a new version of the *roscontrol-sot* package. Indeed, our control scheme needs no more to communicate directly with the robot but with the PAL robotics controller, which implements different functions and formulations. One of the major difficulties is that the proprietary code source is not available; we only had access to its C++ headers and some basic tutorials. Developing this interface to keep all the functionalities implemented in the *roscontrol-sot* package (for instance, to keep the recording of the logs and creating all the necessary signals needed by the SoT in the *dynamic-graph* structure), took us months of work (including the following remark).

Moreover, as the robot has a modified operating system called Ferrum (equivalent to Ubuntu), we created a Docker Merkel (2014) container to have exactly the same environment as the one on the robot to test our codes. Installing the SoT packages on this environment was not trivial as some packages had conflicting dependencies with the PAL robotics packages. Finally, we succeeded to test in this Docker container our interface and our torque controller using the PAL simulator available on Ferrum. An additional difficulty is that the simulator renders the behavior at a rate five times slower than the reality. Then, a small and slow oscillation in the simulator is, in fact, a high-frequency one in reality and can lead to dangerous behaviors.

One has to note that in the study by Dantec et al. (2021), the MPC is not embedded on the robot and is interfaced with the PAL robotics low-level controller *via* a ROS topic. This simpler choice was made because it is a stand-alone package (no SoT or dynamic-graph framework) and does not send commands at high frequency (200 Hz). ROS topics may induce latency and do not allow to send high-frequency commands, leading to real-time issues.

#### 7.3.2 Experiment Results on a Posture Task

Once we achieve satisfying results on the PAL robotics simulator, we tested the classical formulation of our torque controller using inverse dynamics on the real robot on a simple postural task. The task weights and gains used are presented in Tables 9, 10, as the “Fail” experiment.

**TABLE 9:** Set of tasks for the torque control scheme on the posture task.

Tasks	Priority	Weight
Feet contacts	0	—
Posture regularization in half-sitting	I	10

**TABLE 10:** Task gains of the torque control scheme for the posture task.

Experiments	Gains	Legs	Torso
Fail	*K*_p*posture*_	(800, 800, 800, 800, 800, 800)	(1,000, 1,000)
Success	*K*_p*posture*_	(50, 50, 50, 50, 50, 50)	(100, 100)
—	—	Arms	Head
Fail	*K*_p*posture*_	(800, 800, 800, 800, 800, 800, 800, 800)	(100, 100)
Success	*K*_p*posture*_	(50, 50, 50, 50, 50, 50, 50, 50)	(10, 10)

**FIGURE 12:**
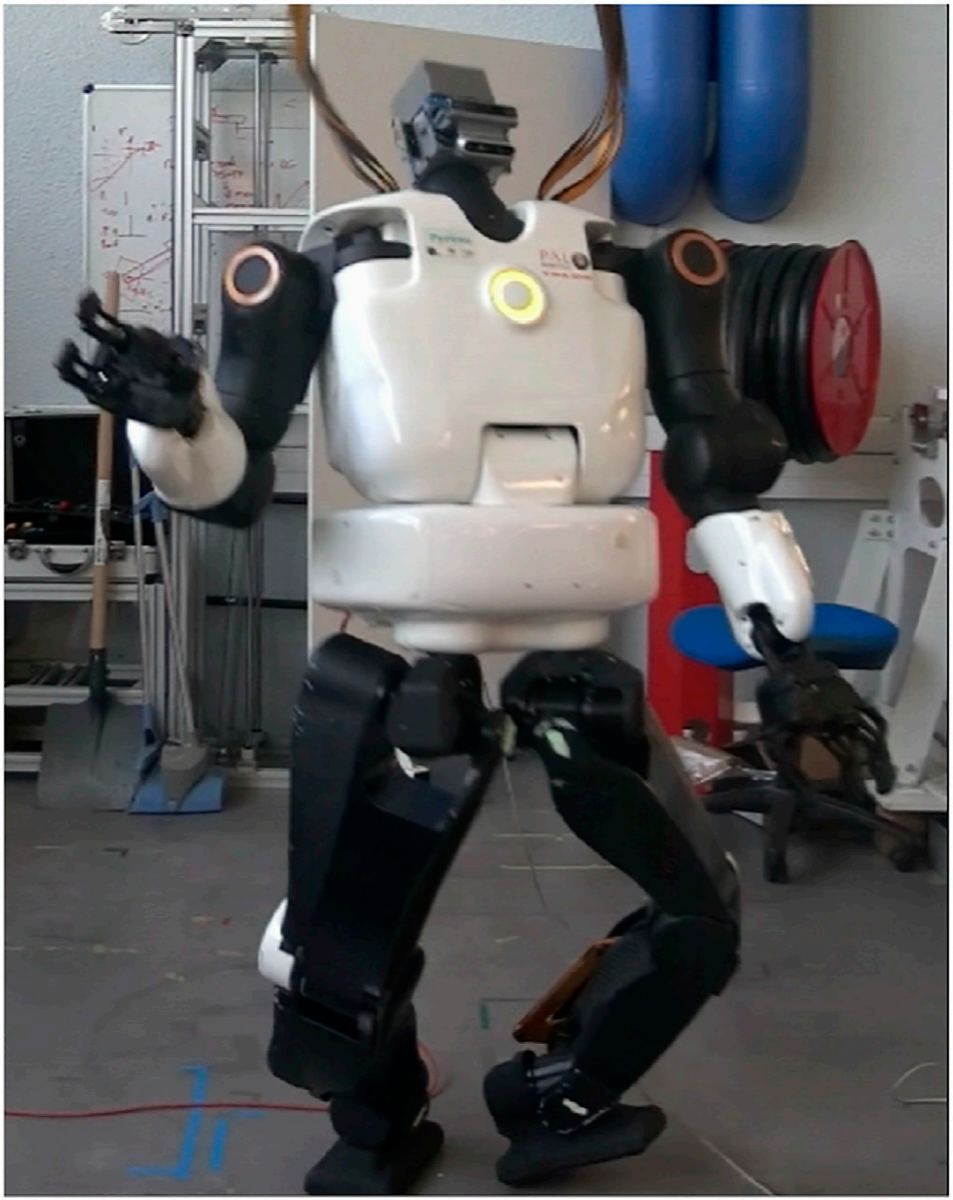
Failed experiment using torque control for a postural task.

After few repetitions of a sinusoidal motion on the robot arm, the system diverged brutally and blocked two of its harmonic drives: the waist and the right shoulder (we pushed the emergency button, but the robot had the time to reach the harmonic drive blocks). Figure 12 presents the result failure. After investigation, it seems that the gains tuned in simulation (which simulates the actuation chains) were too high for the real robot. Thus, tuning the gains even on a proper simulator with the model of the actuators is not enough to ensure the safety of the solution. We know that some tuning is always necessary on the real robot, but we wrongly assumed that the solution would remain quite stable. Thus, to provide a safe and reliable interaction with the environment and possibly humans, we have looked for a way to ensure system stability. The video of the failed experiment is available at this link: https://peertube.laas.fr/videos/watch/31fa2562-ba13-4043-a996-c2b8d5b21f4a. Unfortunately, it was not possible to repair the robot at the laboratory because the right shoulder and the torso were preventing the back cover of the robot to be removed (which needed to be removed to access the shoulder harmonic drive). Thus, the robot had to be sent back to PAL robotics for repair.

**FIGURE 13:**
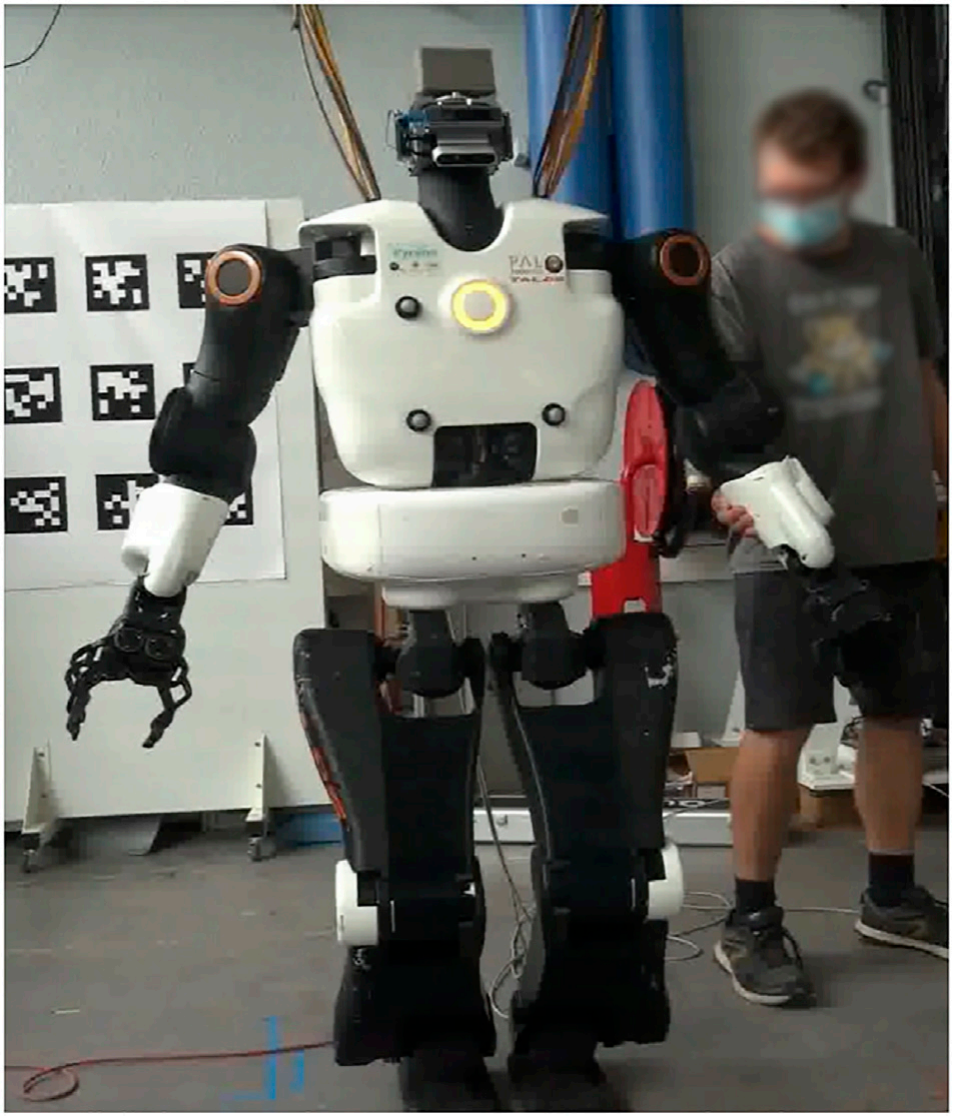
Successful experiment using torque control for a postural task.

By lowering the gain value on the posture task, as presented in Table 10, we succeeded to have a stable and compliant behavior of the robot. A video demonstrating this compliant behavior is available at the following link: https://peertube.laas.fr/videos/watch/e9d8948d-08d5-4de9-8f42-2986fbbf0242 and depicted by Figure 13. At the end of the video, the robot falls because the contacts on the feet have been disturbed (the feet moved), breaking the constraint of the QP.

This small success encouraged us to add the CoM task for further tests. Unfortunately, this task does not work on the real robot. Instead of correcting the CoM error, the QP seems to make it divergent. It is a behavior that does not appear in the simulator where the experiment is carried out. After investigations, this problem may arise due to imprecise calibration or identification of the robot. The difficulty of performing this procedure once the robot is assembled is to excite the parameters to be identified. For instance, part of the torso is particularly difficult to manipulate to observe the variables to be identified. It was the starting point of another research work outside the scope of this study.

## Conclusion

The contribution of this article is the benchmarking of three whole-body control implementations on the commercially available humanoid robot TALOS. Two of them are position-based (with DCM and CoM admittance control): a lexicographic QP using inverse kinematics and a WQP using TSID with an AM task. The last one is a WQP using TSID in torque with an AM task. They are evaluated in Gazebo on flat, uneven terrains, and climbing stairs on the criterion of trajectory tracking, energy consumption, passivity, and computational cost.

In general, both position control schemes present the same results with less energy consumption and higher passivity for the *TSID position* controller. A better tuning of the tasks gains may improve its results on the ZMP tracking.

On the other hand, the *TSID torque* controller shows better results in terms of smoothness of the trajectory tracking, energy consumption, and passivity of the walk without impacts and can achieve a 60-cm walk with steps of 1 s in simulation. This confirms the high capabilities of a torque control scheme coupled with angular momentum regularization (see, for instance, Atlas in DARPA robotics challenge Koolen et al. (2016)). In average, the *TSID* controllers reach 1 kHz of the control loop necessary for real-time control; nonetheless, the *IK* scheme has the best computational time.

For our future studies, we plan to control the hip flexibility of TALOS so that we can evaluate the three controllers on the real robot. Moreover, it would be interesting to compare the controllers on different robotic platforms.

## Data Availability

The original contributions presented in the study are included in the article/Supplementary Material; further inquiries can be directed to the corresponding author.
